# Nano-Delivery of a Novel Inhibitor of Polynucleotide Kinase/Phosphatase (PNKP) for Targeted Sensitization of Colorectal Cancer to Radiation-Induced DNA Damage

**DOI:** 10.3389/fonc.2021.772920

**Published:** 2021-12-23

**Authors:** Sams M. A. Sadat, Melinda Wuest, Igor M. Paiva, Sirazum Munira, Nasim Sarrami, Forughalsadat Sanaee, Xiaoyan Yang, Marco Paladino, Ziyad Binkhathlan, Feridoun Karimi-Busheri, Gary R. Martin, Frank R. Jirik, David Murray, Armin M. Gamper, Dennis G. Hall, Michael Weinfeld, Afsaneh Lavasanifar

**Affiliations:** ^1^ Faculty of Pharmacy and Pharmaceutical Sciences, University of Alberta, Edmonton, AB, Canada; ^2^ Department of Oncology, Cross Cancer Institute, Faculty of Medicine and Dentistry, University of Alberta, Edmonton, AB, Canada; ^3^ Department of Chemistry, Faculty of Science, University of Alberta, Edmonton, AB, Canada; ^4^ Department of Pharmaceutics, College of Pharmacy, King Saud University, Riyadh, Saudi Arabia; ^5^ Department of Biochemistry and Molecular Biology, University of Calgary, Calgary, AB, Canada; ^6^ Department of Medicine, University of Calgary, Calgary, AB, Canada; ^7^ Department of Chemical and Material Engineering, University of Alberta, Edmonton, AB, Canada

**Keywords:** DNA repair, DNA damage, PNKP, radio-sensitization, colorectal cancer, ionizing radiation, nanoparticle, combination therapy

## Abstract

Inhibition of the DNA repair enzyme polynucleotide kinase/phosphatase (PNKP) increases the sensitivity of cancer cells to DNA damage by ionizing radiation (IR). We have developed a novel inhibitor of PNKP, i.e., A83B4C63, as a potential radio-sensitizer for the treatment of solid tumors. Systemic delivery of A83B4C63, however, may sensitize both cancer and normal cells to DNA damaging therapeutics. Preferential delivery of A83B4C63 to solid tumors by nanoparticles (NP) was proposed to reduce potential side effects of this PNKP inhibitor to normal tissue, particularly when combined with DNA damaging therapies. Here, we investigated the radio-sensitizing activity of A83B4C63 encapsulated in NPs (NP/A83) based on methoxy poly(ethylene oxide)-*b*-poly(α-benzyl carboxylate-ε-caprolactone) (mPEO-*b*-PBCL) or solubilized with the aid of Cremophor EL: Ethanol (CE/A83) in human HCT116 colorectal cancer (CRC) models. Levels of γ-H2AX were measured and the biodistribution of CE/A83 and NP/A83 administered intravenously was determined in subcutaneous HCT116 CRC xenografts. The radio-sensitization effect of A83B4C63 was measured following fractionated tumor irradiation using an image-guided Small Animal Radiation Research Platform (SARRP), with 24 h pre-administration of CE/A83 and NP/A83 to Luc^+^/HCT116 bearing mice. Therapeutic effects were analyzed by monitoring tumor growth and functional imaging using Positron Emission Tomography (PET) and [^18^F]-fluoro-3**’**-deoxy-3**’**-L:-fluorothymidine ([^18^F]FLT) as a radiotracer for cell proliferation. The results showed an increased persistence of DNA damage in cells treated with a combination of CE/A83 or NP/A83 and IR compared to those only exposed to IR. Significantly higher tumor growth delay in mice treated with a combination of IR and NP/A83 than those treated with IR plus CE/A83 was observed. [^18^F]FLT PET displayed significant functional changes for tumor proliferation for the drug-loaded NP. This observation was attributed to the higher A83B4C63 levels in the tumors for NP/A83-treated mice compared to those treated with CE/A83. Overall, the results demonstrated a potential for A83B4C63-loaded NP as a novel radio-sensitizer for the treatment of CRC.

**Graphical Abstract:**
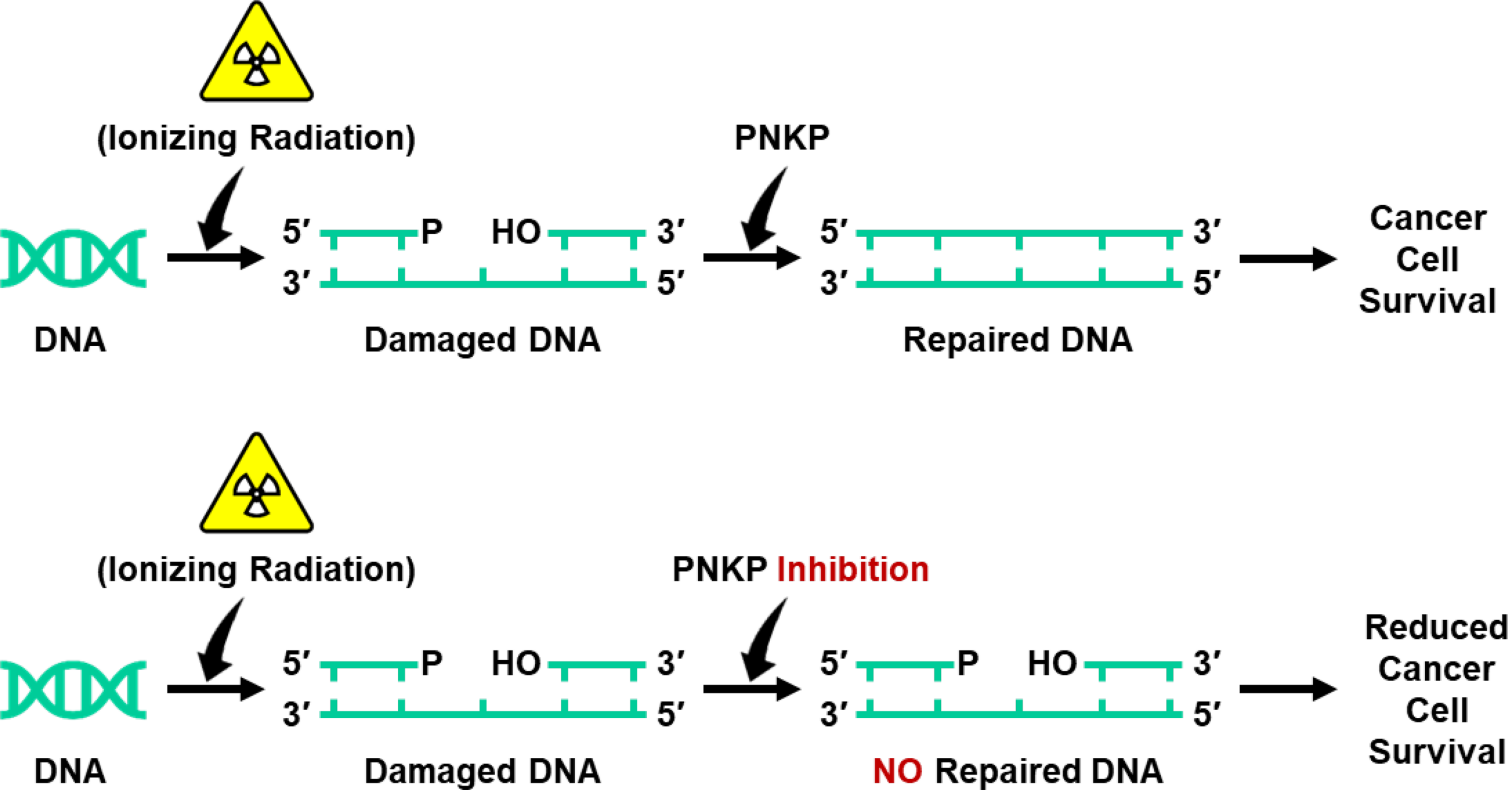


## Introduction

Colorectal cancer (CRC) is the second most common cause of cancer death globally (1) and its incidence is expected to increase by 33% by 2028 (2). Clinical outcomes from the conventional treatment options in CRC appear to depend on the location as well as molecular features of individual tumors (3). Thus, the best treatment decisions must be individualized for patients (4–6). Surgery is a very common option for most CRC patients (7). Adjunctive chemotherapy or ionizing radiation (IR) is often accompanied before or after surgery. Although IR is not a preferred option to treat colon cancer, it is fairly common in rectal cancer (7). Radiation therapy, often with neoadjuvant chemotherapy, is considered to help in shrinking the localized CRC tumors before surgery (8, 9). Radiation therapy may also be used to eradicate cancer cells that may have been left behind with the resection boundary after the surgery (10).

Inherent or acquired cellular resistance mechanisms in CRC cells can undermine the effectiveness of IR, eventually leading to cancer recurrence in CRC patients (11–13). IR generates DNA strand breaks. However, the intracellular capacity to repair damaged DNA is one of the major causes of resistance to IR (12, 14). Inhibition of DNA repair is considered a promising approach to improve the sensitivity of cancer cells to IR, thus, different DNA repair enzymes have been validated as therapeutic targets for radiosensitization in various cancers (15–22).

Human polynucleotide kinase-phosphatase (PNKP) is identified as a key enzyme involved in DNA repair following damage by IR or topoisomerase I inhibitors (e.g. irinotecan) in many types of cancer including CRC (23–26). PNKP phosphorylates DNA 5’-termini and dephosphorylates DNA 3’-termini, which allows DNA polymerases and ligases to rejoin the damaged strands of the DNA. The validity of PNKP as a therapeutic target in sensitizing cancer cells to topoisomerase I inhibitors and IR, has been previously shown by our research team and others (26–30). Through RNAi screening, we made the exciting discovery that the deficiency of a tumor suppressor protein, i.e., phosphatase and TENsin homolog (PTEN), makes cancer cells even more sensitive to the PNKP inhibition (31, 32). This has inspired the development of small molecule inhibitors of PNKP by our research team.

A83B4C63 is a second generation polysubstituted imidopiperidine small molecule inhibitor of PNKP with IC_50_ and K_D_ values in the low micro and nanomolar range, respectively (33). The water-solubility of A83B4C63 is <1 mM and its log D value is ~4.16, which makes this compound a non-ideal candidate for the drug development process. To overcome the limitation of poor water-solubility, and at the same time reduce the access and radio/chemo-sensitizing effects of A83B4C63 in normal tissues, we have developed NP formulations of this compound, which were based on methoxy poly(ethylene oxide)-poly(α-benzyl carboxylate-ε;-caprolactone) (mPEO-*b*-PBCL). Passive targeting of solid tumors by NPs is attributed to the presence of leaky vasculature as well as impaired drainage of the lymphatic system at the tumor site (34–42). The nanocarriers of appropriate size (below 200 nm) and specific surface properties can extravasate from the leaky vasculature at the tumor sites, while the impaired lymphatic drainage prevents their rapid removal out of the tumor microenvironment (43, 44). This phenomenon, which is known as the enhanced permeation and retention (EPR) effect, is believed to play a key role in preferential distribution of nanocarriers in solid tumors compared to many normal tissues (45–48). In a recent study, we have shown that polymeric micellar NPs (PMNPs), formed through self-assembly of poly(ethylene oxide)-blockpoly(α-benzyl carboxylate-ϵ-caprolactone) (PEO-*b*-PBCL) containing methoxy-PEO (mPEO) or acetal-PEO (acPEO), and radiolabeled with ^64^Cu resulted in a 3-fold increased measurable accumulation into subcutaneous HCT116 tumors (perhaps due to the EPR effect) versus muscle tissue as determined with PET (49).

In our previous studies, the nano-formulation of A83B4C63 was shown to effectively reduce the viability of PTEN-deficient CRC, as monotherapy (33). The mPEO-*b*-PBCL based NPs of A83B4C63 were also shown to sensitize CRC cells to IR and irinotecan, *in vitro* (24). *In vivo*, the NPs of A83B4C63 were tolerated better than conventional formulations of this compound and showed significantly enhanced delivery and activity of incorporated A83B4C63 in PTEN-deficient HCT116 xenografts in mice. The objective of the current study was to assess the therapeutic effect of conventional versus mPEO-*b*-PBCL nano-formulations of A83B4C63 for sensitization of wild type CRC models to IR, both *in vitro* and *in vivo*.

## Materials and Methods

### Materials

Methoxy polyethylene oxide (mPEO) (average molecular weight of 5000 g/mol), Cremophor EL: Ethanol (CE), and all research grade organic solvents were purchased from Sigma (St. Louis, MO, USA). α-Benzyl carboxylate-ε-caprolactone monomer was synthesized by Alberta Research Chemicals Inc. (Edmonton, AB, Canada). Stannous octoate was purchased from MP Biomedicals Inc. (Tuttlingen, Germany).

### Synthesis of A83B4C63 and PEO-*b*-PBCL Copolymer

The polysubstituted imidopiperidine compound, A83B4C63, was synthesized using a three-component aza[4 + 2]/allylboration reaction and purified to homogeneity *via* reverse-phase HPLC as previously described (50). The structure of the compound was confirmed by NMR, infrared spectroscopy, and LC-MS as previously reported (24).

**Figure 1:**
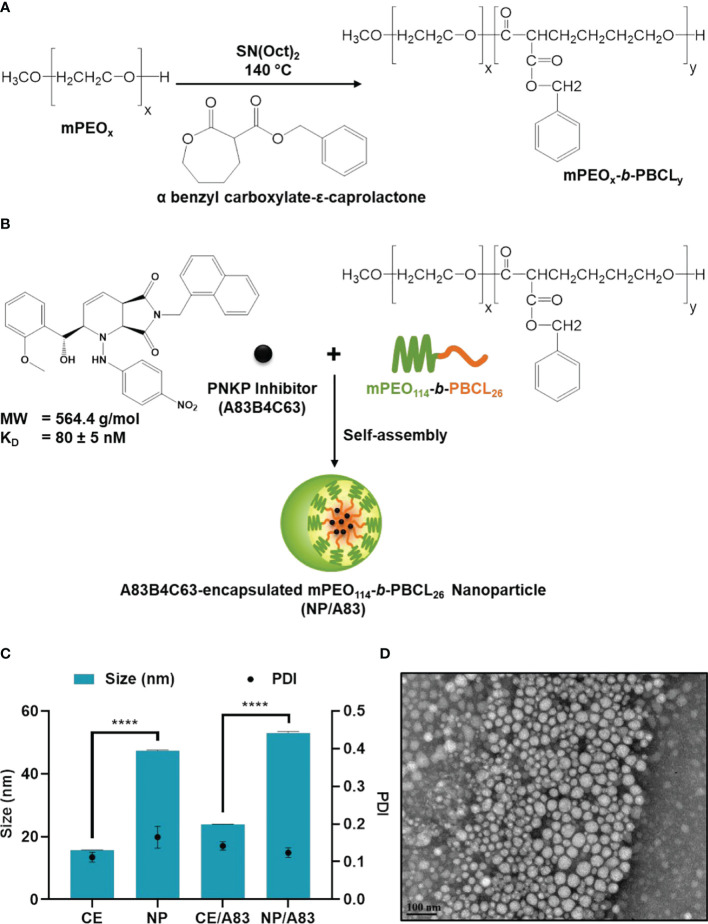
Chemical structure of **(A)** methoxy poly(ethylene oxide)-*b*-poly(α-benzyl carboxylate-ε-caprolactone or mPEO-*b*-PBCL and **(B)** illustration of encapsulation process of 2-[hydroxy(2-methoxyphenyl)methyl]-6-(naphthalene-1-ylmethyl)-1-[(4-nitrophenyl)amino]-2H, 4aH, 7aH-pyrrolo[3,4-b]pyridine-5,7-dione or A83B4C63. **(C)** Physicochemical characterization of water-soluble CE, empty NP, A83B4C63-solubilized (CE/A83), and A83B4C63-encapsulated mPEO-*b*-PBCL (NP/A83) formulations (n = 10). Hydrodynamic diameter and polydispersity index (PDI) of NP/A83 micelles in aqueous medium were obtained using dynamic light scattering (DLS). **(D)** TEM image of A83B4C63-encapsulated micellar formulation (NP/A83) in aqueous medium. The TEM image was obtained at a magnification of 110,000X at 75 kV. The bar in the bottom left corner of the image indicates a scale of 100 nm. Data from three independent experiments were compared by two-way ANOVA multiple comparison test following Tukey’s method. (****p ≤ 0.0001). The TEM image displayed is a representative of at least three independent experiments.

The mPEO-*b*-PBCL block copolymer with 26 degree of polymerization (DP), i.e., the number of repeating units in a polymer chain, for the PBCL block was synthesized by ring-opening polymerization of α-benzyl carboxylate-ε-caprolactone using mPEO (MW: 5000 g/mol) as an initiator and stannous octoate as catalyst according to the method described previously (24, 51) (**Figure 1**). The synthesized copolymers were characterized for their average molecular weights by ^1^H NMR (600 MHz Avance III - Bruker, East Milton, ON, Canada) using deuterated chloroform (CDCl_3_) as solvent and tetramethylsilane as an internal reference standard.

### Formulation and Characterization of A83B4C63-Encapsulated mPEO-*b*-PBCL NPs Versus A83B4C63 Solubilized With the Aid of CE

A83B4C63-encapsulated mPEO-*b*-PBCL NPs (NP/A83) were prepared as previously described (24). In brief, 10 mg A83B4C63 and 30 mg mPEO-*b*-PBCL polymer were completely dissolved in 1 mL of acetone. Then, the organic phase was transferred dropwise to 10 mL aqueous phase and left overnight with continuous stirring with a magnetic bar in a fume hood to completely evaporate the organic solvent. The unencapsulated A83B4C63 was removed by centrifugation at 11600 × g for 5 min to obtain NP/A83. The NP/A83 solution was then transferred into Amicon Ultra-15 centrifugal filter tubes (molecular weight cut-off, 100 kDa; Millipore, ON, Canada) and centrifuged at 11600 × g for 20 min at 4°C in order to concentrate as required. CE/A83 formulation was prepared by previously described method (33). In brief, 2 mg of A83B4C63 drug was dissolved in 400 μL of 100% ethanol to prepare the oil phase using a water bath sonicator until the drug was completely dissolved. Then, 400 μL of CE solution was added into it and vortexed for 2-3 min. The oil phase was poured in water phase (5% dextrose in double distilled water) to emulsify the solubilized drug in the form of NP (**Figure S1**) and was purified using a 0.22 μm syringe filter. The average size and polydispersity index (PDI) of the NP and CE formulations were measured by dynamic light scattering (DLS) using a Malvern Zetasizer 3000 (Malvern Instruments Ltd, Malvern, UK). A83B4C63 loading and encapsulation efficiency were measured and analyzed using a Varian Prostar 210 HPLC system. Reversed phase chromatography was carried out with a Microsorb-MV 5 μm C18-100 Å column (4.6 mm × 250 mm) with 20 μL of sample injected and eluted under isocratic conditions with a solution of 0.1% trifluroacetic acid/acetonitrile (1:1 v/v) at a flow rate of 0.7 mL/min at room temperature. Detection was performed at 280 nm wavelength for A83B4C63 using a Varian 335 Photodiode Array HPLC detector (Varian Inc., Palo Alto, CA, USA). In this study, A83B4C63 control was solubilized with DMSO for all *in vitro* experiments, while for *in vivo* experiments, A83B4C63 was dissolved with the aid of CE (CE/A83). Finally, the A83B4C63 loading and loading efficiency were calculated according to the following equations:


$$\text{A}83\text{B}4\text{C}63\text{ loading }(\%)=\frac{\text{Weight of the encapsulated A}83\text{B}4\text{C}63\text{ in NPs}}{\text{Total weight of the polymer in NPs}}\times 100$$

$$\text{A}83\text{B}4\text{C}63\text{ encapsulation efficiency }(\%)=\frac{\text{Weight of the encapsulated A}83\text{B}4\text{C}63}{\text{Initial weight of the A}83\text{B}4\text{C}63\text{ added}}\times 100$$

### Transmission Electron Microscopy (TEM)

The morphology of self-assembled structures under study was investigated by TEM using a Morgagni TEM (Field Emission Inc., Hillsboro, OR, USA) with Gatan digital camera (Gatan, Pleasanton, CA, USA). In brief, 20 μL of micellar solution with a polymer concentration of 0.25 mg/mL or Cremophor EL at a concentration of 0.2 mg/mL was placed on a copper-coated grid. The grid was held horizontally for 1-2 min to allow the colloidal particles to settle down. The excess fluid was removed by filter paper. The copper-coated grids holding the aqueous samples were then negatively stained by 2% phosphotungstic acid. After 2 min, the excess fluid was removed by filter paper and the grid was loaded into the TEM for image analysis.

### Cell Lines

Wild type HCT116 cells were obtained from the American Type Culture Collection and luciferase positive Luc^+^/HCT116 cells were generated as previously described (52). Cells were routinely cultured at 37°C in 5% CO_2_ in a humidified incubator in a 1:1 mixture of Dulbecco’s modified Eagle medium and F12 (DMEM/F12) supplemented with 10% FBS, 50 U/mL penicillin, 50 mg/mL streptomycin, 2 mmol/L L-glutamine, 0.1 mmol/L nonessential amino acids, and 1 mmol/L sodium pyruvate. All culture supplements were purchased from Invitrogen (Burlington, ON, Canada).

### Microscopic Study for γ-H2AX Evaluations

1 × 10^5^ wild type HCT116 cells were seeded onto each glass coverslip in a 35-mm Petri dish and incubated overnight to attach. The cells were then pretreated with the nano-formulations for 24 h prior to 3 Gy γ-irradiation. Irradiation was carried out at room temperature at a dose rate of 0.66 Gy/min. After irradiation, the cells were incubated for two time points up to 6 h. After the incubation, the cells were fixed with 4% paraformaldehyde in PBS for 20 min, then permeabilized, and blocked with 1% BSA in 1 x PBS/0.1% Tween 20 for 20 min. After 3 washes with 1 x PBST, anti-phospho-histone H2A.X (Ser139) antibody (catalog# 05-636, Millipore, Temecula, CA, USA) at 1:4000 dilution was applied to the cells and incubated for 1 h at room temperature. The cells were washed three times with 1 x PBST and then incubated with Alexa Fluor 488 goat anti-mouse secondary antibody (catalog# A11059, Life Technologies, Carlsbad, CA, USA) at a 1:200 dilution in 0.1% BSA/1 x PBST for 1 h in the dark. After washing the cells, the coverslips were mounted on the slides with DAPI-containing mounting media (53) (Molecular Probes, Eugene, Oregon, USA) at 1 µg/mL concentration. Images were taken with an Axio Imager Z2 microscope (Carl Zeiss, Jena, Germany) using MetaMorph 7 and MetaXpress 6 software (Molecular Devices, San Jose, CA, USA) to image and quantify foci.

### Western Blot

Western blot was used to assess the level of cleaved caspase 3/7 and PARP induced by A83B4C63 as free drug (CE/A83) and NP (NP/A83) formulation in HCT116 cells with or without radiation. Initially, 1.5 million cells were plated. Then, cells were treated with CE/A83 and NP/A83, or vehicles alone, at an A83B4C63 concentration of 10 µM, or equivalent drug free CE and NP levels. After 24 h incubation with A83 formulations or vehicle controls, cells were exposed to a fixed dose of radiation (4 Gy) using a ^60^Co Gamma irradiator (AECL, Chalk River, ON, Canada). The cells were harvested at either 1 or 4 h after exposure to IR. Each experiment was performed in triplicate.

Protein extracts for western blot analysis were prepared using commercial RIPA lysis buffer (ThermoFisher Scientific, Mississauga, ON, Canada) supplemented with a cocktail of protease inhibitors (Millipore Sigma, Mississauga, ON, Canada). Protein concentrations were measured using the BCA assay kit (Pierce/ThermoFisher Scientific, Mississauga, ON, Canada) according to the manufacturer’s protocol. Equal concentrations of protein were separated by SDS-PAGE and transferred to nitrocellulose membranes. After blocking with 5% skimmed milk in TBST (50 mM Tris-HCl, pH 7.4, 150 mM NaCl, and 0.1% Tween 20), the blots were incubated with the respective primary antibodies (caspase-3 catalog# 9662S, caspase-7 catalog# 9492S, PARP catalog# 9542S) and secondary antibody (HRP-linked anti-rabbit IgG cat# 7074S) purchased from Cell Signaling Technology (Whitby, ON, Canada). The protein bands were detected using an enhanced chemiluminescence (ECL) based system (Pierce/ThermoFisher Scientific, Mississauga, ON, Canada) and quantified by densitometric analysis using ImageJ software.

### Xenograft Models

NIH-III nude mice were purchased from Charles River Laboratories (Wilmington, MA, USA). All animal studies were conducted in accordance with the guidelines of the Canadian Council on Animal Care and with approval from the local Animal Care Committee of the Cross Cancer Institute (Edmonton, AB, Canada). The HCT116 and Luc^+^/HCT116 xenograft tumor mouse models were generated by subcutaneous injection of 0.5 × 10^6^ cells in a 100 µL mixture of culture media and matrigel matrix (Corning, MA, USA) (1:1 v/v) in the right flank or left shoulder of 4 - 6 week-old female NIH-III nude mice. The CRC cell implanted mice were routinely monitored for tumor growth and signs of sickness. Animals reaching early endpoints as set in our animal protocol were euthanized. All animals were euthanized at day-22 following the tumor inoculation.

### *In Vivo* Anticancer Activity of Combination Therapies

This study was performed on Luc^+^/HCT116 xenografts developed as described above. When the tumor volume reached 80 to 150 mm^3^, mice were randomly assigned into test groups receiving empty NP without IR (n = 6), or empty NP (n = 6), CE/A83 (n = 6), and NP/A83 (n = 7) formulations of A83B4C63 with a fractionated radiation dose of 3 x 5 Gy q.a.d. The treatments (empty NPs, PNKP inhibitor A83B4C63 alone or CE/A83, A83B4C63-encapsulated NPs (NP/A83) were started on day 0. On day -2 (2 days before starting the treatments), tumor sizes were measured with a digital slide caliper and by bioluminescence using an *in vivo* imaging system (IVIS^®^). All drugs were given *via* intravenous (IV) injection *via* tail vein and administered on days 0, 2, and 4. The IV A83B4C63 dose was 25 mg/kg, which was injected three times one day apart. Mice received three fractionated radiation doses of 5 Gy every alternative day. The excipient dose, i.e., empty NP in control groups was selected equivalent to their amounts in the NP/A83 test group. The length (L) and width (W) of the tumor were measured two times per week and the tumor volume (TV) was calculated using the formula TV = (L × W^2^)/2. The measurements continued until day 22 (since the day of tumor inoculation) when all mice were euthanized.

The fractionated radiation therapy using a daily dose of 5 Gy was started on day 1 and given 3x including days 3 and 5. Radiation therapy was administered using the image-guided small-animal radiation research platform (SARRP; Xstrahl Inc. Suwanee, GA, USA) Mice were placed ventrally onto the bed of the SARRP and immobilized with continuous isoflurane with anesthesia. A cone beam computed tomography (CT) scan was acquired first for each mouse and used for radiation therapy planning per mouse using integrated Muriplan/Murislice^®^ software (Xstrahl Medical & Life Sciences, Camberley, UK). The radiation target volume was defined as the tumor volume contoured from the cone beam CT scan and the isocenter defined in the center of the tumor volume and radiation doses were calculated. After therapy planning, the radiation therapy to the target tumor area was delivered using a 0.15 mm copper filter with 220 kVp X-rays and 13 mA using and two opposing dorsal beams at 45 to 60 degrees and minus 45 to 60 degrees and a 10 mm x 10 mm square-shaped collimator at a dose rate of 0.042 Gy/sec and an exposure time of 60 s per beam. The collimator size was big enough to completely cover tumor tissue for the applied irradiation.

### *In Vivo* Imaging Systems (IVIS^®^) for Evaluating Anticancer Activity of CE/A83 and NP/A83 With or Without Radiation

The animals inoculated with Luc^+^/HCT116 and treated as described above were also imaged for the expression of luciferase to follow their tumor growth. For the optical imaging, mice were subcutaneously injected with the XenoLight D-Luciferin - K+ salt bioluminescent substrate (PerkinElmer, UK) at a dose of 10 µL/g of body weight before the luciferase detection. Mice were anesthetized and placed in the dark chamber of a IVIS^®^ LUMINA XMRS optical imaging systems (PerkinElmer, Waltham, MA, USA) for whole-body animal imaging and the emitted photons were quantified and analyzed using Living Image^®^ Software (PerkinElmer, Waltham, MA, USA). Imaging of live animals was performed twice a week.

### PET Imaging

Luc^+^/HCT116 tumor-bearing female NIH-III nude mice from the radiation therapy study (as described above) were analyzed on days 10-12 after last treatment for tumor proliferation using Positron Emission Tomography (PET). Mice were anesthetized by isoflurane (100% O_2_). A needle catheter was placed into the tail vein of these mice and 3 - 6 MBq of [^18^F]FLT in 100 to 150 µL saline were injected. [^18^F]FLT was synthesized at the cyclotron research facility of the Cross Cancer Institute according to the previously described procedure (54) using 5-O-(4,4-dimethoxytrityl)-2,3-anhydrothymidine (ABX GmbH, Radeberg, Germany) as the synthesis precursor. Radioactivity in the injection solution in a 0.5 mL insulin syringe was determined using a dose calibrator (AtomlabTM 300, Biodex Medical Systems, Shirley, NY, USA). After radiotracer injection, mice were allowed to regain consciousness for about 40 to 45 min before anesthetizing them again. They were immobilized in prone position into the center field of view of a preclinical INVEON^®^ PET scanner (Siemens Preclinical Solutions, Knoxville, TN, USA). Acquisition data were collected in three-dimensional list mode for 10 min, reaching ~60 min post injection. Static PET images were reconstructed using a maximum *a posteriori* (MAP) algorithm. Image files were further processed using the ROVER v2.0.51 software (ABX GmbH, Radeberg, Germany). Masks defining three-dimensional regions of interest (ROI) over tumor tissue were defined and ROI’s were analyzed with 50% threshold of radioactivity uptake. Mean standardized uptake values [SUV*_mean_* = (measured radioactivity in the ROI/mL tumor tissue)/(total injected radioactivity/mouse body weight)] were calculated for each ROI.

### Biodistribution of CE/A83 and NP/A83 Formulations in HCT116 Tumor-Bearing Mice

The biodistribution profiles of A83B4C63 in CE and NP forms were assessed in wild type HCT116 tumor-bearing NIH-III mice. Tumor-bearing mice were developed as described above, except for the use of HCT116 cells instead of Luc^+^/HCT116. When the tumor volume reached 1200 to 1500 mm^3^, mice were randomly assigned and grouped into three test groups (n = 3). The test groups received CE/A83 or its NP form three times, one day apart at an IV dose of 25 mg/kg. The control mice received empty NPs. 4, 24, and 48 h after the last injection, all mice were euthanized, and blood, excised tumors and other organs including brain, heart, lung, liver, kidney, and spleen were collected to define drug levels using an LC/MS/MS method of quantification as previously described (33). In brief, all snap-frozen dissected tumor tissues were weighed and homogenized with an ice-cooled solution of acetonitrile/water (50:50 v/v) using an electric hand homogenizer. The collected whole blood samples of the mice were centrifuged at 2000 × g for 5 min at 4°C to separate the plasma. Tissue homogenate samples were centrifuged at 2000 × g for 15 min at 4°C. To 250 μL of plasma/homogenized tissues 1000 µL cold acetonitrile was added. The mixture was vortexed for 5 min and then the samples were centrifuged at 2000 × g for 20 min. The solutions were separated and transferred to clean tubes and evaporated to dryness.

An Agilent 1100 HPLC system coupled to a Waters Quattro Micro triple quadrupole mass spectrometer (Waters, Milford, MA, USA) and attached to an Agilent Poroshell 120 SB- C18 2.7-micron LC column with dimensions of 2.1 mm x 50 mm was used. The column was heated to 35°C. The mobile phase consisted of water with 0.1% formic acid (A) and acetonitrile with 0.1% formic acid (B). A gradient elution was programmed to commence with 20% B for post-injection followed by a gradual increase in 3 min of B to 95%. The composition was maintained for 3 min when it was gradually decreased back to 20% of B in 0.1 min. The flow rate was 0.3 mL/min and 2 µL of sample was injected. Standard curves were linear over the range of 1 - 1000 ng/mL (r^2^ > 0.99; coefficient of variation < 20%). The lowest limit of quantification was set at 1 ng/mL. The mass spectrometer was operated in positive mode with capillary voltage at 3.2 kV, source temperature at 120°C, desolvation temperature at 275°C, and desolvation gas flow at 800 L/h. Instrumental control and data analysis were performed using MassLynx software (Waters, Milford, MA, USA).

Propranolol dissolved in the solution of acetonitrile/water with 50:50 v/v ratios was used as an internal standard. The dried residues in sample vials were reconstituted with 100 µL of internal standard solution with vigorous vortexing before placing into the auto-sampler of the LC/MS/MS (Waters Quattro Micro ± ES MS Triple Quadrupole, Milford, MA, USA) fitted with an Agilent Technology: Poroshell 120 SB-C18 2.1x50 mm, 2.7-micron column. The mobile phase consisted of 50:50 v/v ratios of water with 0.1% formic acid and acetonitrile with 0.1% formic acid.

The terminal elimination rate constant was estimated from the log-linear portion of the plasma concentration - time curves. Because of the destructive sampling procedure used for the collection of blood and tissues from different animals at each time point, the area under the plasma/tissue curve (AUC) was estimated using the trapezoidal rule from the average plasma concentrations at different time points and the variance of AUC was estimated using Bailer’s method based on the standard error of the mean (SEM). The ratio of tissue concentration at each time point to that of plasma (Kp) was also calculated and reported.

### Statistical Analysis

GraphPad Prism 9 software (La Jolla, CA, USA) was used for statistical analysis. Significance of differences between groups was assessed using one-way and two-way ANOVA followed by Tukey’s *post-hoc* test, where appropriate. If a significant difference was found among the groups, median ranks between pairs of groups were compared using the Mann-Whitney U test. A value of *p ≤ 0.05* was considered as statistically significant in all experiments.

For biodistribution experiment, the AUC of plasma or tissue versus time curves were obtained using the approach outlined by Bailer (55). Pairwise comparisons of the AUC were performed at α = 0.05. The critical value of Z (Zcrit) for the two-sided test after Bonferroni adjustment was 2.24 (56), and the observed value of Z (Zobs) was calculated as previously described (57, 58). When Zobs values are greater than Zcrit, the difference between AUCs was considered statistically significant.

## Results

### Physicochemical Characterization

The ^1^H NMR spectra and peak assignments of mPEO-*b*-PBCL (**Figure 1A**) and A83B4C63 (**Figure 1B**) were previously reported (24, 51, 59–61). According to the calculations based on the ^1^H NMR spectra, the DP was 26 for PBCL block in mPEO-*b*-PBCL copolymers. A83B4C63 encapsulation into the mPEO-*b*-PBCL micellar NPs was performed following a simple one-step self-assembly nanoprecipitation method (**Figure 1B**). 21.97 ± 0.65% loading and 70.28 ± 3.47% encapsulation efficiency were measured when A83B4C63-encapsulated mPEO-*b*-PBCL NPs (NP/A83) were prepared at a 1:3 w/w A83B4C63:mPEO-*b*-PBCL ratio. The NP/A83 were ~60 nm in diameter on average and showed a low polydispersity index (PDI), i.e., < 0.25, indicating the uniformity of the nanocarrier population in terms of diameter (**Figure 1C**). After A83B4C63-solubilization by CE formulation, the average size of CE/A83 micelles was < 35 nm in diameter, which was significantly lower (****p ≤ 0.0001) than that of NP/A83. However, no significant difference was measured for the PDI values obtained from CE/A83 and NP/A83. The diameter of the empty carriers from both formulation types i.e, NP, CE, were significantly lower (*p ≤ 0.05) than their drug-encapsulated counterparts. As shown in **Figure 1D**, the TEM image confirmed the formation of spherical NP/A83 micelles with uniform size. In the TEM image, a similar distribution pattern in the micellar population having a clear boundary was observed that also indicated the low aggregation tendency among the formed micelles.

### Mechanistic Evaluations

**Figure 2:**
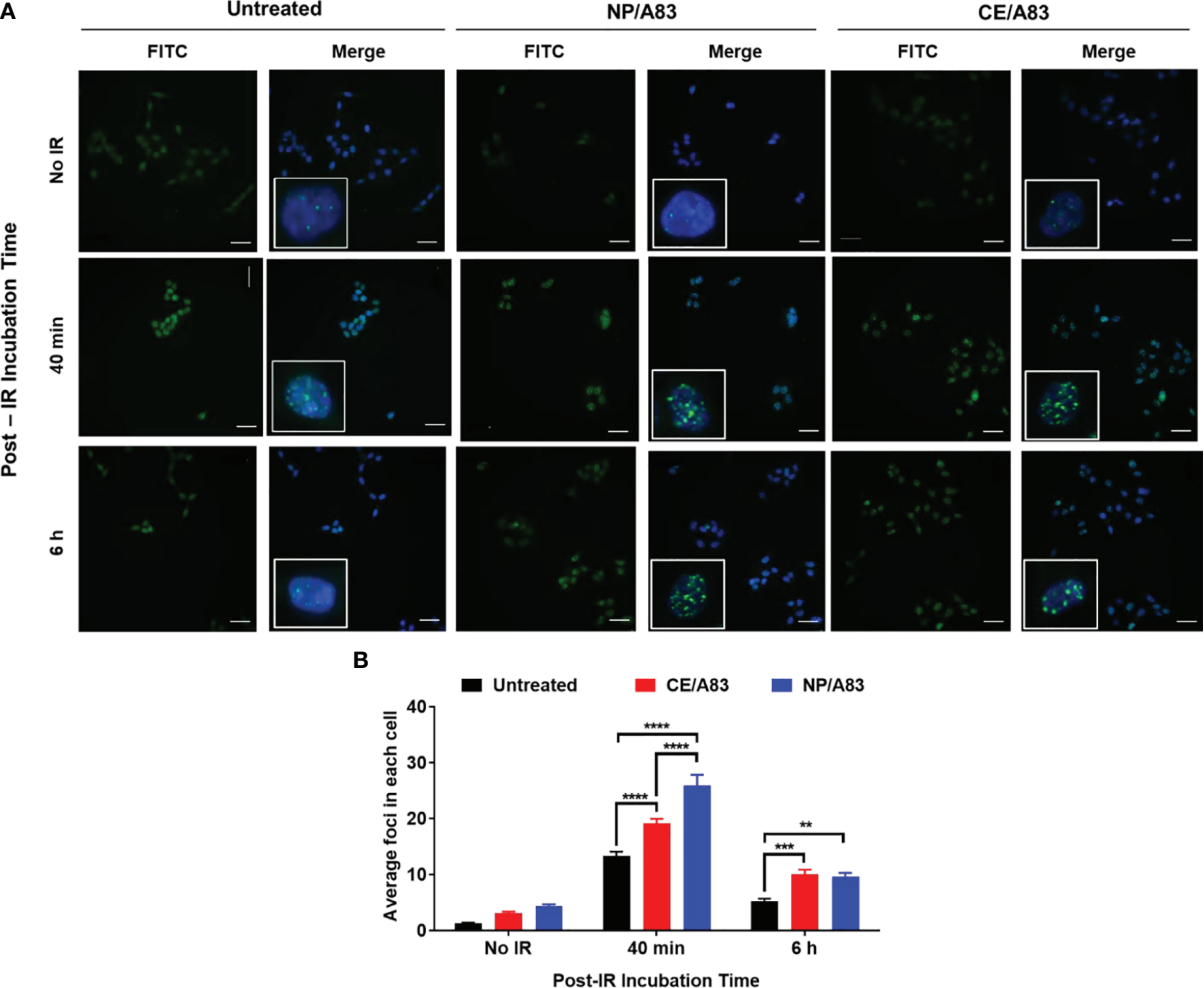
Formation and repair of double strand breaks of DNA analyzed by γ-H2AX foci formation (H2A.X Ser139) in HCT116 cells. **(A)** Representative images of γ-H2AX (green) foci and nuclei (blue) were counterstained with DAPI. Inset figures show typical γ-H2AX foci in individual cells. **(B)** Quantitative analysis for the number of foci in each treated cell. 24 h prior to 3 Gy γ-irradiation, cells on the coverslips were treated with 10 µM CE/A83 and NP/A83. At 40 min or 6 h after irradiation, cells were fixed, permeabilized, and stained for foci to be visualized under the microscope. MetaXpress 6 software was used to take images and to quantify the number of foci in each cell. Data from three independent experiments were compared by two-way ANOVA multiple comparison test following Tukey’s method. Differences were considered significant if (**p ≤ 0.01, ***p ≤ 0.001, and ****p ≤ 0.0001). Micrographs displayed are representative of at least three independent experiments; scale bar = 40 μm.

Upright microscopic evaluations were performed to assess the DNA damage following treatment of cells with a combination of PNKP inhibitor and a fixed dose of IR (3 Gy). **Figure 2A** shows the wide-field fluorescence images of the γ-H2AX-positive cells treated with CE/A83 and NP/A83. Here, we studied the temporal and spatial distribution of the foci of the phosphorylated form of the histone protein H2AX (γ-H2AX) that is known to be modified, upon γ-irradiation, by kinases activated by double-strand breaks in cellular DNA. Qualitative analysis based on the microscopic images of the distribution of foci in each cell indicated greater clustering of DNA damage by radiation in cells when they were pre-treated with A83B4C63 delivered by either CE or NP formulations. Quantitative analysis using the MetaXpress 6 software was performed to quantify the number of foci in each cell. A significantly higher number of γ-H2AX-positive foci was observed 40 min after γ-irradiation in NP/A83-pretreated cells than in CE/A83 pretreated and untreated (radiation alone) groups. The difference observed at 40 min is likely related to the inhibition of repair by A83B4C63 treatment. The number of foci decreased at 6 h post γ-irradiation for both A83B4C63 formulations. Significantly higher foci numbers post γ-irradiation in cells pretreated with either CE/A83 or NP/A83 compared to cells without drug demonstrated the proof of concept for the radio-sensitizing activity of our PNKP inhibitor, i.e., A83B4C63.

We also analyzed the induction of cleaved PARP, cleaved caspase-7, and cleaved caspase-3 expressions following treatment by A83B4C63 with and without radiation. Both CE/A83 and NP/A83 formulations slightly induced the level of cleaved PARP, cleaved caspase-7 and cleaved caspase-3, but the level of induction was low, suggesting that apoptosis does not play a major role in the cellular response to radiation with or without the repair inhibitor (**Figure S2**).

### *In Vivo* Radio-Sensitizing Activity of CE/A83 and NP/A83 in Wild Type HCT116 Xenografted Mice

**Figure 3:**
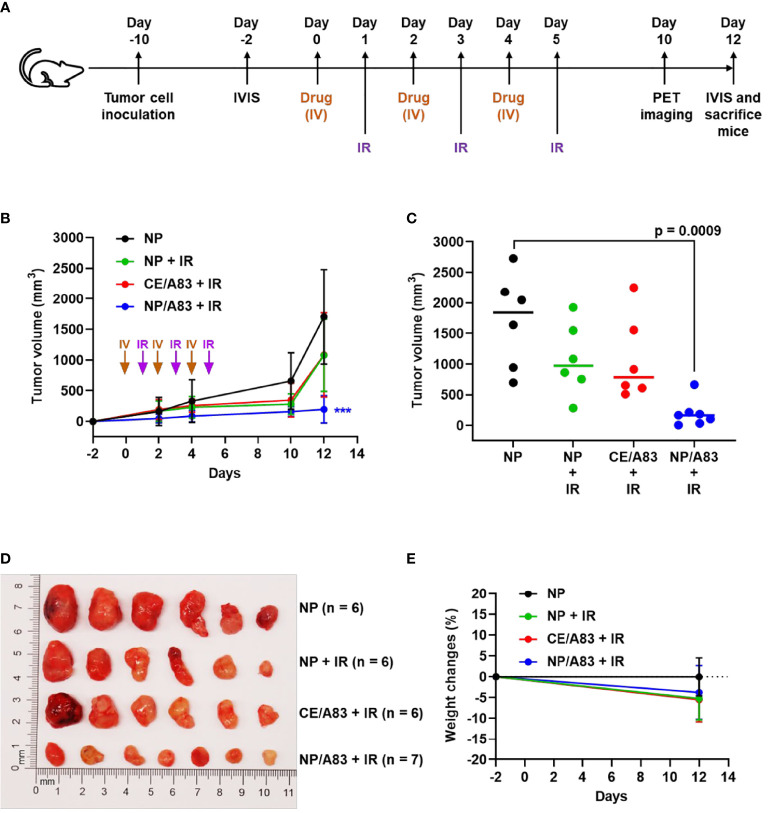
**(A)** Schematic experimental design for evaluating the anticancer activity of A83B4C63 as CE and NP formulations in female NIH-III nude mice following IV administration (n = 6 or 7). Colorectal Luc^+^/HCT116 cells were inoculated and grown as subcutaneous tumor xenografts in the right flank of the mice. When tumors became palpable based on the tumor measurement by calipers the treatments started. The *in vivo* live imaging system (IVIS^®^) was also used before and after treatment to follow tumor growth. A total of 25 mice were divided into 4 groups (6 + 6 + 6 + 7), which were intravenously injected with (i) control empty NPs, (ii) control empty NP plus 3 x 5 Gy IR, (iii) CE/A83 (A83B4C63 formulated with the aid of CE) plus 3 x 5 Gy IR, and (iv) NP/A83 (A83B4C63-encapsulated mPEO_114_-*b*-PBCL_26_ micelles) plus 3 x 5 Gy IR three times with a one day interval at a dose of 25 mg/kg. **(B)** Average tumor volume growth curves for mice in each treatment group for Luc^+^/HCT116 CRC xenograft. **(C)** The average tumor volumes obtained from the treated groups on day-12 post injection. Using digital calipers, the length (L) and width (W) of the tumor mass were measured 2 times per week and the tumor volume (TV) was calculated according to the following formula, TV = (L × W^2^)/2. **(D)** Images of representative tumors from **(B). (E)** The average percentage for the change in body weight of mice bearing Luc^+^/HCT116 xenografts. Differences were considered significant if (***P < 0.001).

To explore the radio-sensitizing anticancer activity of intravenously administered CE/A83 and NP/A83 at a dose of 25 mg/kg three times a week in mice bearing Luc^+^/HCT116 xenografts, all mice were inoculated with 0.5 million cells 10 days (day -10) before the treatment schedule as shown in **Figure 3A**. According to the experimental design (**Figure 3A**), the tumor-bearing control mice received empty NPs in isotonic 5% dextrose. Mice receiving systemic empty NPs plus IR were also used as a control group. To investigate the anticancer activity for this combination treatment approach, we conducted both digital slide caliper measurement and bioluminescence live imaging to monitor the growth of xenograft tumors in the mice.

As shown in **Figure 3B**, the mice receiving empty NP with no IR exhibited rapid CRC tumor growth compared to other treatment groups that received IR. IR induced a growth delay, but mice receiving empty NP plus IR or CE/A83 plus IR still showed moderate tumor size increases. However, NP/A83 plus IR demonstrated the slowest tumor growth among the treatment groups. **Figure 3C** represents the average tumor volumes obtained from the treated groups on day 12 post first IV injection. A highly significant growth delay in the xenografted tumors was observed for the mice receiving NP/A83 plus IR compared to the control (empty NP without IR).

As shown in **Figure 3C**, the decrease in the average size of excised tumors from NP/A83-treated mice matched the average tumor volumes obtained from either slide caliper or bioluminescence measurements. At the day of termination (day 12), the average tumor volumes reached 1706.02 ± 773.80, 1076.45 ± 586.78, and 1082.72 ± 685.81 mm^3^ (n = 6), in the mice treated with empty NP, empty NP plus IR, and CE/A83 plus IR, respectively, whereas the tumor volumes remained as low as 196.56 ± 221.01 mm^3^ (n = 7) in mice treated with IV NP/A83 plus IR. The overall results clearly showed the *in vivo* radio-sensitizing activity of A83B4C63 in its NP formulation in wild type Luc^+^/HCT116 CRC xenografts in mice, which was in contrast to no statistically significant activity for the CE formulation of this PNKP inhibitor compared to control groups receiving empty NPs with or without IR (*p* > 0.05). **Figure 3D** also shows the images of excised tumors from the mice of all treatment groups at the termination day. These data verified the results of tumor growth measurement by the digital slide calipers (and IVIS^®^, see below). The measured mean body weight variation of the mice receiving systemic treatments were within a 20% margin (**Figure 3E**) and did not show any statistical difference irrespective of the treatment groups.

**Figure 4:**
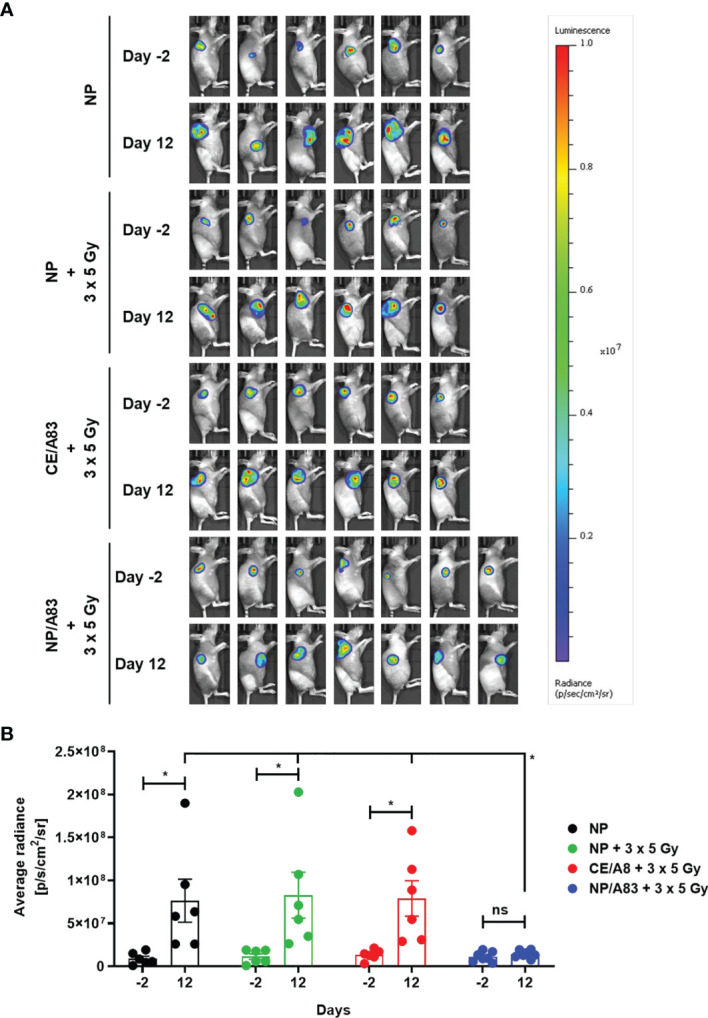
**(A)** Representative bioluminescence images from the tumor-bearing mice on days-2 and 12 for evaluating the radio-sensitizing anticancer activity of A83B4C63 as CE and NP formulations in female NIH-III nude mice following IV administration (n = 6 or 7). 0.5 × 10^6^ colorectal Luc^+^/HCT116 cells were inoculated and grown as subcutaneous tumor xenografts in the right flank of the female athymic NIH-III nude mice. When tumors became palpable, a total of 25 mice were randomly assigned into 4 groups (6 + 6 + 6 + 7), which were intravenously injected with (i) control empty NPs, (ii) control empty NP plus 3 x 5 Gy IR, (iii) CE/A83 (A83B4C63 formulated with the aid of CE) plus 3 x 5 Gy IR, and (iv) NP/A83 (A83B4C63-encapsulated mPEO_114_-*b*-PBCL_26_ micelles) plus 3 x 5 Gy IR three times with a one day interval at a dose of 25 mg/kg. The mice were imaged for luciferase intensity 2 days before the treatment started. Radiation therapy was administered using an image-guided SARRP platform. **(B)** Quantitative analysis for the average radiance (photons per s per cm^2^ per square) bioluminescence signal for the four treatment groups of mice on day -2 (2 days prior to start treatment) and day 12 (termination day). To show tumor growth, the tumor radiance at day -2 (two days before treatment) is subtracted from tumor radiance at day 12 from the same mouse. Differences were considered significant if *p ≤ 0.05. ns stands for not significant.

To further evaluate the radio-sensitizing anticancer activity of A83B4C63, tumor growth in mice was also detected by *in vivo* bioluminescence imaging. Based on the average radiance for bioluminescence of Luc^+^/HCT116 cells in mice (**Figure 4A**), NP/A83 pretreatment with fractionated IR dose of 3 x 5 Gy was found to delay the tumor growth significantly when compared to the other treatment groups. At day 12 (**Figure 4B**), the quantitative analysis exhibited a significant difference in average radiance in the NP/A83-pretreated group (*p ≤ 0.05, two-way ANOVA) in comparison to that of other pretreatment groups, including the empty NP, empty NP plus IR, and CE/A83 plus IR cohorts. Therefore, the radiance for bioluminescence of Luc^+^/HCT116 xenografts in the respective treatment groups of mice showed a similar pattern in tumor growth to that observed by slide caliper measurements. When comparison was made between the day -2 (2 days prior to starting treatments) and day 12 (termination day), significant increases in luciferase-tagged cancer cells (bioluminescence) were found for all treatment groups except NP/A83-treated mice, which did not show any difference in the bioluminescence of xenografts from day -2 to day 12. The data validated the anti-tumoral activity of systemic NP/A83 administration in the HCT116 CRC xenograft model as a novel radio-sensitizing nanomedicine.

### PET Imaging of HCT116 CRC Xenograft Mice

**Figure 5:**
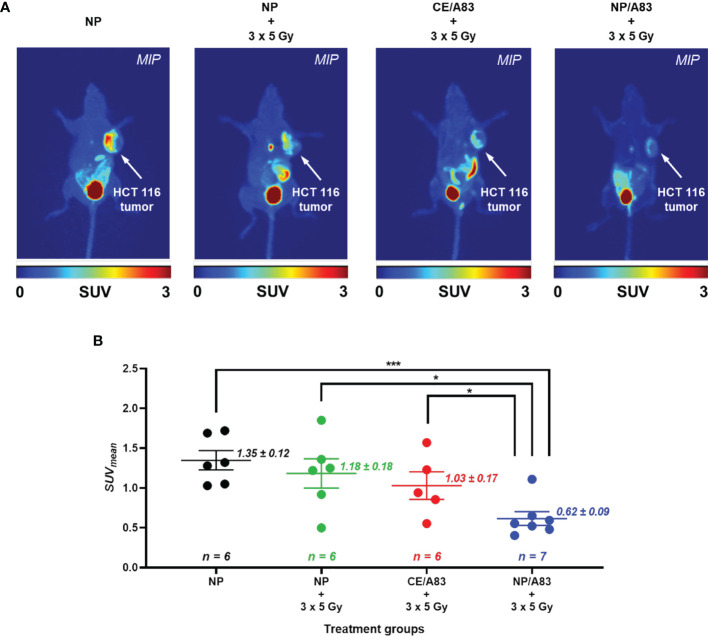
**(A)** Static [^18^F]FLT-PET images after 60 min post injection of female athymic NIH-III nude mice (one representative image from each treatment group) post treatment (day 10) with empty NP, CE/A83, and NP/A83 with a fractionated 3 x 5 Gy dose of radiation. The control mice received empty NP without radiation. The white arrows indicate the xenograft CRC. **(B)** The quantitative data for the analyzed SUV*_mean_* values of the [^18^F]FLT tumor uptake. Differences were considered significant if *p ≤ 0.05, and ***p ≤ 0.001 following two-way ANOVA followed by Tukey’s method. Data are shown as mean ± SEM from n experiments.

**Figure 5** summarizes results from the non-invasive PET imaging experiments using [^18^F]FLT to determine the tumor proliferation *in vivo* in highly multiplying cancer cells in the HCT116 xenografts to determine the radio-sensitizing activity of A83B4C63 formulations in these tumors. In line with the results of caliper and bioluminescence measurements, administration of NP/A83 at 25 mg/kg dose plus IR led to a significant reduction of [^18^F]FLT uptake in the HCT116 xenografts. This contrasted with CE/A83 plus IR that did not show any significant reduction of [^18^F]FLT uptake when compared to the control receiving empty NPs plus IR. Following tumor uptake levels of [^18^F]FLT were determined as mean standardized uptake values (SUV*_mean_*) ± SEM: 1.35 ± 0.12, 1.18 ± 0.18, 1.03 ± 0.17 (all n = 6) and 0.62 ± 0.09 (n = 7), for empty NP, empty NP plus IR, CE/A83 plus IR, and NP/A83 plus IR, respectively. When compared for significant differences as shown in **Figure 5B**, the NP/A83 plus 3 x 5 Gy IR-treated mice group displayed a significantly lower SUV*_mean_* value than that of empty NP without IR at α level of 0.001. The difference in SUV*_mean_* value for mice that received NP/A83 plus IR was significantly lower than for the mice that received empty NPs or CE/A83 plus IR (*p < 0.05). However, no significant differences were observed between the NP plus IR and CE/A83 plus IR treatment groups.

### Biodistribution Profile of CE and NP Formulations of A83B4C63

**Figure 6:**
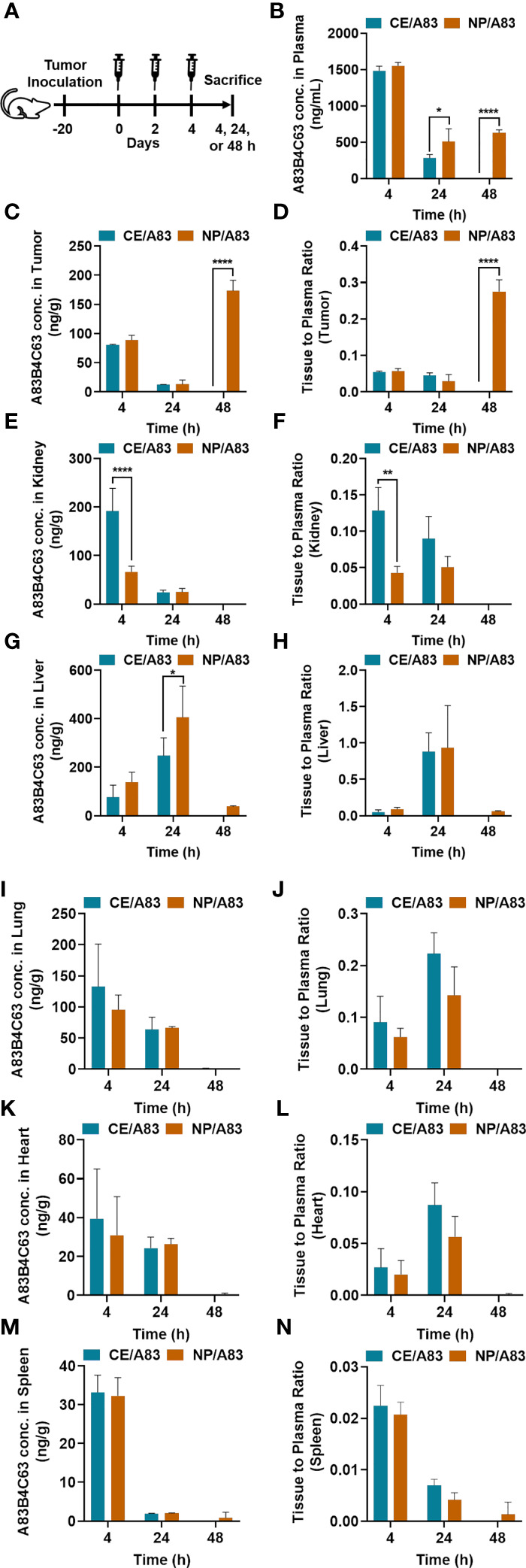
**(A)** The experimental schedule for determining the bio-fate of A83B4C63 intravenously delivered *via* CE and NP formulations in CRC tumor-bearing mice. **(B–N)** The biodistribution profile of A83B4C63 in wild-type HCT116 CRC xenograft bearing NIH-III female nude mice (n = 3) 4, 24, and 48 h after tail vein administration of CE/A83 and NP/A83 formulations. Mice were inoculated with HCT116 CRC cells. 21 days following tumor cell inoculation, the mice received CE/A83 and NP/A83 formulations intravenously at a dose of 25 mg/kg three times with a one-day interval. The control mice received empty NPs, equivalent to the amounts used in the test groups. 4, 24, and 48 h after the last IV injection, all mice were euthanized to collect blood plasma by cardiac puncture. Then, tumors and other organs including kidney, liver, lung, heart, and spleen were collected, snap frozen in liquid nitrogen, and stored at -80°C for later use. Drug concentration was quantified using LC/MS/MS (mean ± SD). **(B)** A83B4C63 plasma concentration versus time curves of CE/A83 and NP/A83 formulations in HCT116 xenograft tumor-bearing mice. **(C**, **E**, **G**, **I**, **K**, **M)** represent A83B4C63 concentrations obtained from the excised tumor, kidney, liver, lung, heart, and spleen, respectively, after administration of CE/A8 and NP/A83. **(D**, **F**, **H**, **J**, **L**, **N)** represents the ratio of tissues (tumor, kidney, liver, lung, heart, and spleen, respectively) to plasma concentration of CE/A83 and NP/A83-treated xenograft mice. Differences were considered significant if *p ≤ 0.05, **p ≤ 0.01, and ****p ≤ 0.0001 following two-way ANOVA followed by Tukey’s test.

**Figure 6** and **Table 1** represent the plasma or tissue concentration versus times profile, as well as tissue to plasma ratio of A83B4C63 formulations and their AUC following IV administration of the above formulations at a dose of 25 mg/kg three times in mice bearing HCT116 tumors (**Figure 6A**). As shown in **Figure 6B**, the concentration of A83B4C63 obtained by CE/A83 formulation fell below the limit of detection after 24 h while NP/A83 formulation yielded plasma drug concentrations (****p ≤ 0.0001) significantly above the detection limits for up to 48 h. The concentration of A83B4C63 obtained by NP/A83 formulation was significantly higher at 24 h (*p ≤ 0.05) and 48 h (****p ≤ 0.0001) when compared with that of CE/A83. This resulted in a significantly higher plasma AUC level for the mice that received NP/A83 (34246.64 ± 3710.36) treatment to those that received CE/A83 (21078.86 ± 1534.31) (*p ≤ 0.05, student’s t-test).

**Table 1:** Calculated area under the curve (AUC) for plasma concentrations of CE/A83 and NP/A83 formulations in HCT116 tumor-bearing mice until 48 h time point post drug administration.

Specimens	Formulations	AUC ± SEM (ng.h/mL or g)
		
Plasma	CE/A83	21078.86 ± 1534.31
	NP/A83	34246.64 ± 3710.36*
Tumor	CE/A83	1071.11 ± 21.00
	NP/A83	3254.89 ± 259.94*
Kidney	CE/A83	2455.59 ± 374.71*
	NP/A83	1211.89 ± 177.67
Liver	CE/A83	6198.00 ± 2032.99
	NP/A83	10773.38 ± 3161.52*
Lung	CE/A83	2740.85 ± 695.96
	NP/A83	2409.93 ± 249.46
Heart	CE/A83	931.07 ± 188.99
	NP/A83	894.02 ± 161.52
Spleen	CE/A83	374.87 ± 45.43
	NP/A83	376.25 ± 49.65

Significant differences between CE/A83 and NP/A83 were distinguished by *p < 0.05 (n = 3) according to student’s t-test.

Biodistribution data (**Table 1** **)** showed significantly higher AUC values for NP/A83 than for CE/A83 in tumor and liver, while the AUC of NP/A83 was lower in kidney compared to CE/A83 (*p ≤ 0.5, student’s t-test). However, no significant differences were observed between these treatment groups in lung, heart, and spleen **(** **Figures 6E–M** **)**.

A83B4C63 concentrations in excised tumors from the mice were also measured and the results are shown in **Figures 6C, D**. The results showed tumor accumulation of A83B4C63 delivered by NP/A83 formulation at 48 h post injection, whereas the detected concentrations of A83B4C63 in CE/A83-treated xenografts were below the limit of detection at this time point. Calculation of tumor to plasma concentration ratio for the two formulations showed a significant increase at 48 h for the NP formulation as well.

Notably, CE/A83 formulation resulted in significantly higher accumulation of A83B4C63 in the kidney at 4 h post dose time point compared to that of NP/A83 (****p ≤ 0.0001) (**Figure 6E**). Similarly, the kidney to plasma ratio (Kp value) yielded a significantly higher ratio for A83B4C63 in the kidney of mice treated with CE/A83 (**p ≤ 0.01) compared to that of NP/A83-treated mice. In contrast, the obtained A83B4C63 concentration was significantly higher in the liver samples (**Figures 6G, H**) of NP/A83-treated mice than that of CE/A83-treated mice at 24 h (*p ≤ 0.05) post dose, only. However, no significant difference was observed in liver to plasma ratio between CE/A83 and NP/A83 treatment groups.

## Discussion

Human PNKP phosphorylates DNA 5´-termini and dephosphorylates DNA 3´-termini, allowing DNA polymerases and ligases to rejoin the strands, and therefore plays a key role in both single- and double-strand break repair (30). PNKP has been identified as a potential therapeutic target in different types of cancer, as depletion of PNKP in cancer cells or tumor xenografts has shown a synthetic lethal partnership with the loss of the tumor suppressor protein PTEN (24, 33). Moreover, the downregulation of PNKP by siRNA or its inhibition by small molecule inhibitors have been shown to sensitize cancer cells to IR and to topoisomerase I inhibitors (24–26, 31, 32).

We have identified new small molecule inhibitors of PNKP. Our initial attention was on inhibition of the DNA 3´-phosphatase activity of PNKP, with a polysubstituted imidopiperidine, A12B4C3, identified as the first hit (31, 32). At a non-cytotoxic dose, A12B4C3 effectively sensitized human lung cancer A549 cells to IR and camptothecin. However, it failed to further sensitize the cancer cells that were already depleted of PNKP by shRNA, providing strong evidence for PNKP as the druggable target of A12B4C3 (25). The Reilly group showed that A12B4C3 sensitizes human myeloid leukemia cells to radio-immunotherapy providing more evidence for the promise of PNKP inhibitors as radio-sensitizers (62, 63).

PNKP inhibitors render tumors more susceptible to DNA damage by IR or topoisomerase I inhibitors but may act similarly on normal cells leading to intolerable toxicities in patients. To overcome the problem of non-specificity for cancer and, at the same time, to enhance the solubility of PNKP inhibitors for *in vivo* administration, we have developed NP formulations of a second generation polysubstituted imidopiperidine, named A83B4C63. Nanocarriers can significantly improve the therapeutic index of anticancer agents (43, 44). Nanocarriers are small enough to enter leaky blood vessels in solid tumors, but not normal blood vessels (64). Lymphatic function in tumors is impaired, thus nanocarriers are not drained effectively and accumulate in the tumor (65–67). NPs have the capacity to deliver higher quantities of drugs to targets and can be actively targeted to tumor cells (48). Nanocarriers of conventional anticancer agents (e.g., doxorubicin, paclitaxel, and irinotecan) have already found their way into the clinic (68, 69).

At a concentration range of 1-10 μM, both free and encapsulated A83B4C63 in PEO-*b*-PBCL NPs were effective in reducing the viability of PTEN^-/-^ HCT116 cells but did not affect wild-type (WT) or HCT116/PTEN^+/+^ cell viability (24, 33). Our previous study has also shown the success of PEO-*b*-PBCL NP formulations of A83B4C63 as monotherapeutic in the selective inhibition of tumor growth in PTEN-deficient HCT116 tumor xenografts, due to synthetic lethality in this cancer model (33). This contrasted with the CE formulations of this drug candidate that did not show anticancer activity in HCT116/PTEN^-/-^ tumor xenografts when compared to mice receiving 5% dextrose. The current study focused on *in vitro* and *in vivo* evaluation of NP versus CE formulations of A83B4C63 in sensitization of HCT116/PTEN^+/+^ tumors to IR. Radiation therapy is commonly used to treat rectal cancer (7, 70). In colon cancer, radiation therapy, is mostly used as a neoadjuvant therapy before surgery or as an adjuvant therapy after or during surgery to further eradicate cancerous cells (8–10). Radiation therapy is also used in metastatic CRC, where cancer has spread to liver or lung (22).

The NP/A83 formulation can successfully be reproduced and showed an average particle size of < 60 nm with low PDI, consistent with our previous reports (24, 33). The NP/A83 formulation enhanced the solubility of A83B4C63 in water to a level over 6.5 mg/mL, enabling administration of the compound to mice at the desired therapeutic doses (71). Comparisons were made with a conventional CE-based solubilizing formulation of A83B4C63. CE is a well-known water-soluble nanocarrier for cyclosporin A and paclitaxel (72–74). However, CE is associated with acute or chronic side-effects (e.g. anaphylaxis, nephro- and neurotoxicity) (75, 76) and is also known to interfere with the pharmacokinetics of several drugs (77–83).

*In vitro* studies on HCT116 cells revealed the activity of A83B4C63 either as CE or NP formulation in delaying DNA repair and enhancing DNA damage persistence. This was evidenced through the measurement of γ-H2AX foci formation, which showed an increase in foci numbers upon co-treatment of cells with IR plus both A83B4C63 formulations compared to the IR treatment alone (**Figure 2**). The A83B4C63 formulations on their own, without IR, did not cause any significant rise in the level of γ-H2AX foci at the dose applied here, reflecting the lack of DNA damage induced by A83B4C63 alone.

For the *in vivo* studies, a relatively low fractionated dose (3 x 5 Gy) of IR was used to avoid potential side-effects on normal tissues surrounding the irradiated site (84). The treatment groups were shown to be safe and well-tolerated as there was no evidence for any toxicity symptoms, such as weight reduction in mice during and after the treatments. The HCT116 xenografts showed significant tumor growth delay when NP/A83 treatment was combined with the fractionated dose of IR. This observation was similar to our findings of the anticancer effect of A83B4C63 as a synthetic lethal mono-therapeutic in PTEN-deficient HCT116 xenografts, in which only NP/A83 and not CE/A83 was shown to be an effective anticancer agent. The activity of NP/A83 as a radio-sensitizer was confirmed through the analysis of three different tumor parameters: classical tumor volume measurements using slide calipers (**Figure 3**), optical imaging of LUC^+^ tumors (**Figure 4**) and functional PET imaging using [^18^F]FLT (**Figure 5**) to measure proliferation of tumor cells in live animals. Collectively these data validated the intravenously administered NP/A83 as a more effective radio-sensitizer than CE/A83 in CRC xenografts in mice. The data showed the overall lower effectiveness of the CE/A83 formulation in radio-sensitizing activity, *in vivo*. In addition, the data confirmed that [^18^F]FLT PET could be used as a non-invasive functional imaging tool to detect and monitor therapeutic effects of NP/A83 in a translational clinical setting.

To shed some light on the reason behind the superior activity of NP/A83 over CE/A83 *in vivo*, we investigated the biodistribution profile of A83B4C63 in HCT116 CRC tumor-bearing mice following a similar administration schedule as used in the anticancer activity study. A83B4C63 is a new investigational drug and the effect of CE on its pharmacokinetic profile is not known. Our data on the biodistribution of NP/A83 versus CE/A83 formulations at 4, 24, and 48 h post last injection, revealed an interesting pattern (**Figure 6**): In plasma, following the administration of the CE formulation, A83B4C63 was eliminated rapidly, and no detectable drug levels were identified at the 48 h time point. The NP/A83, on the other hand, enhanced the resident time of A83B4C63 in plasma. This profile coincided with a delayed accumulation of A83B4C63 in tumor tissue 48 h following the last dose. Accordingly, a significant enhancement in the AUC of A83B4C63 in tumor tissue for the NP over CE formulation was achieved. This pattern contrasted with the distribution profile of NP versus CE formulations of A83B4C63 in normal tissues, where a decline in drug levels for both formulations was seen from 24 to 48 h. Among the normal tissues, liver was the only organ that showed significantly higher AUC for the NP formulation of A83B4C63. On the other hand, the AUC of NP formulations of A83B4C63 showed reduction in kidneys compared to the CE formulation. The reason for the delayed accumulation of A83B4C63 by its NP formulation in HCT116 xenografts is not clear and needs further investigation. Nevertheless, a sustained inhibition of PNKP resulting from higher accumulation of its nano-formulation in tumor xenografts along with a continuous release of the drug in the tumor site might have been responsible for the higher activity of NP/A83 over CE/A83, *in vivo*. The delayed distribution of NP/A83 in tumor tissue may provide opportunities for the optimization of intervals between chemo or radiation co-treatments with NP/A83, which will be explored in future. In this regard, assessing the variation in the concentration of NP/A83 in the tumor between injections would also be of immense interest.

## Conclusions

In summary, our data demonstrated that the PNKP inhibitor, A83B4C63 loaded into mPEO-*b*-PBCL nanocarriers leads to additional radio-sensitizing effects in a CRC model, as analyzed both *in vitro* and *in vivo*. The present data provide a strong case for potential benefit of nanotechnology in the formulation of drug candidates for clinical development during the drug development process which can be monitored with non-invasive imaging methodologies through their translational path.

## Data Availability Statement

The raw data supporting the conclusions of this article will be made available by the authors, without undue reservation.

## Ethics Statement

All animal studies were conducted in accordance with the guidelines of the Canadian Council on Animal Care and with approval from the local Animal Care Committee of the Cross Cancer Institute and University of Alberta (Edmonton, AB, Canada).

## Author Contributions

SS has been responsible for the design and completion of all studies in this manuscript. He has prepared the first draft of the manuscript. MWu has assisted in the conduction of *in vivo* radiation studies and edited the manuscript. IP, SM, NS, FS, and ZB have provided assistance in the biodistribution studies. XY has provided assistance in fluorescent microscopy measurements. GM, FJ, DM, and AG provided feedback on *in vivo* studies and edited the manuscript. MP has synthesized A83B4C63. DH has supervised the synthesis of A83B4C63 and edited the manuscript. MWe and AL have supervised the study and edited the manuscript. All authors contributed to the article and approved the submitted version.

## Funding

This work was supported by grants funded by the Canadian Institutes of Health Research (MOP 15385) to MWe and (159757 and 178028) to AL, the Alberta Cancer Foundation Transformative Program Project (26603) to DH, FJ, AL, and MWe. Funding from Nanomedicine Innovation Network (NMIN) grant (2019-T1-06) to DH, MWe, and AL is also acknowledged.

## Conflict of Interest

The authors declare the following competing financial interest(s): Material in this manuscript has been included in recent US patent applications. AL is Vice-President of Meros Polymers which has the license to mPEO-b-PBCL polymer used in this manuscript.

## Publisher’s Note

All claims expressed in this article are solely those of the authors and do not necessarily represent those of their affiliated organizations, or those of the publisher, the editors and the reviewers. Any product that may be evaluated in this article, or claim that may be made by its manufacturer, is not guaranteed or endorsed by the publisher.

## Supplementary Material

Supplementary Figure 1
**(A)** Chemical structure of Cremophor EL used in CE/A83 formulation. **(B)** TEM image of CE/A83.

Supplementary Figure 2
**(A)** Representative western blot detection of cleaved PARP, cleaved caspase-7, and cleaved caspase-3 in HCT116 CRC cells pretreated with or without PNKP inhibitor (either CE/A83 or NP/A83 formulation). β-actin was used as a loading control. The conditions for all sample preparations and western blots were the same. The statistical analysis for the protein levels of **(B)** cleaved PARP, **(C)** cleaved caspase-7, and **(D)** cleaved caspase-3 was performed after normalization to β-actin. Differences were considered significant if **p* ≤ 0.05, ***p* ≤ 0.01, ****p* ≤ 0.001, and *****p* ≤ 0.0001 following two-way ANOVA followed by Tukey’s method. Data are expressed as mean ± SD (n = 3).

## Abbreviations

AUC, area under the curve; CDCl_3_, deuterated chloroform; CE, cremophor EL: ethanol; CE/A83, A83B4C63-solubilized cremophor EL: ethanol formulation; C_max_, peak plasma concentration; CRC, colorectal cancer; DLS, dynamic light scattering; DMEM/F12, dulbecco’s modified eagle medium and F12; DNA, deoxy ribonucleic acid; HLB, hydrophilic-lipophilic balance; IR, ionizing radiation; IV, intravenous; IVIS^®^, *in vivo* imaging systems; Kp, tissue to plasma ratio; MAP, maximum a posteriori; mPEO, methoxy polyethylene oxide; mPEO-*b*-PBCL, methoxy poly(ethylene oxide)-*b*-poly(α-benzyl carboxylate-ϵ-caprolactone); MRT, mean residence time; MW, molecular weight; NP, nanoparticle; NP/A83, A83B4C63-encapsulated mPEO-*b*-PBCL nanoparticle; PARP, poly (ADP-ribose) polymerase; PDI, polydispersity index; PET, positron emission tomography; PNKP, polynucleotide kinase/phosphatase; PTEN, phosphatase and TENsin homolog deleted on chromosome 10; ROI, regions of interest; SARRP, small animal radiation research platform; SUV, standardized uptake values; TEM, transmission electron microscopy; T_max_, peak plasma concentration time; TV, tumor volume.
