# Supplementary material for: Nano-Delivery of a Novel Inhibitor of Polynucleotide Kinase/Phosphatase (PNKP) for Targeted Sensitization of Colorectal Cancer to Radiation-Induced DNA Damage
Source: Front Oncol. 2021 Dec 23;11:772920. doi: 10.3389/fonc.2021.772920 (PMC8733593; doi:10.3389/fonc.2021.772920)
Supplement: Supplementary Figure 1 — (A) Chemical structure of Cremophor EL used in CE/A83 formulation. (B) TEM image of CE/A83. [file DataSheet_1.pdf]

Support information

Nano-delivery of a novel inhibitor of polynucleotide kinase/phosphatase (PNKP) for targeted sensitization of colorectal cancer to radiation-induced DNA damage

Sams M. A. Sadat[1], Melinda Wuest[2], Igor M. Paiva[1], Sirazum Munira[1], Nasim Sarrami[1], Forughalsadat Sanaee[1], Xiaoyan Yang[2], Marco Paladino[3], Ziyad Binkhathlan[1, 4], Feridoun Karimi-Busheri[2], Gary R. Martin[5], Frank R. Jirik[5, 6], David Murray[2], Armin M. Gamper[2], Dennis G. Hall[3], Michael Weinfeld[2 *], and Afsaneh Lavasanifar[1, 7 *]

[1] Faculty of Pharmacy and Pharmaceutical Sciences, University of Alberta, Edmonton, AB, Canada

[2] Department of Oncology, Cross Cancer Institute, Faculty of Medicine and Dentistry, University of Alberta, Edmonton, AB, Canada

[3] Department of Chemistry, Faculty of Science, University of Alberta, Edmonton, AB, Canada

[4] Department of Pharmaceutics, College of Pharmacy, King Saud University, P.O. Box 2457, Riyadh 11451, Saudi Arabia

[5] Department of Biochemistry and Molecular Biology, and Department of Medicine, University of Calgary, Calgary, AB, Canada

[6] Department of Medicine, University of Calgary, Calgary, AB, Canada

[7] Department of Chemical and Material Engineering, University of Alberta, Edmonton, AB, Canada

[*] Corresponding authors:

Afsaneh Lavasanifar, Pharm D, PhD

Faculty of Pharmacy and Pharmaceutical Sciences, Edmonton, Alberta T6G 2E1, Canada

Tel: +17804922742, Fax: +17804921217, E-mail: afsaneh@ualberta.ca

Michael Weinfeld, PhD

Department of Oncology, Cross Cancer Institute, Faculty of Medicine and Dentistry, University of Alberta, Edmonton, Alberta T6G 1Z2, Canada

Tel: +17804328438, Fax: +17804328428, E-mail: mweinfel@ualberta.ca

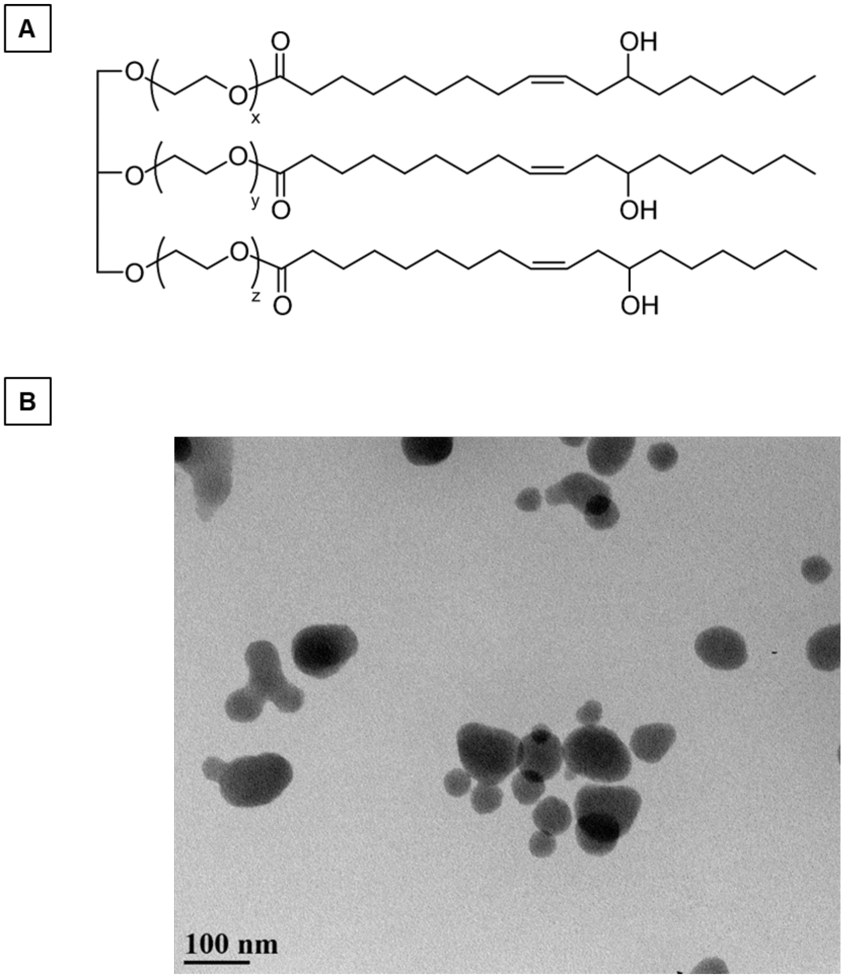

Figure S1- A) Chemical structure of Cremophor EL used in CE/A83 formulation. B) TEM image of CE/A83.

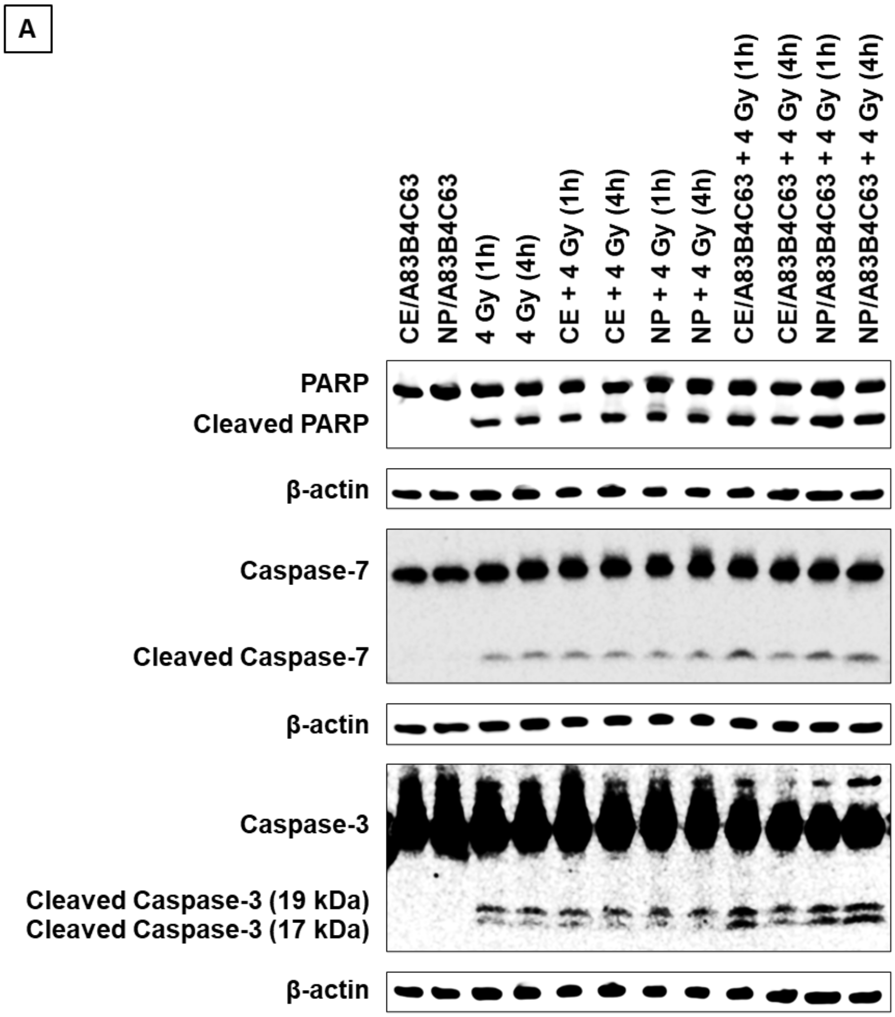
A
CE/A83B4C63
NP/A83B4C63
4 Gy (1h)
4 Gy (4h)
CE + 4 Gy (1h)
CE + 4 Gy (4h)
NP + 4 Gy (1h)
NP + 4 Gy (4h)
CE/A83B4C63 + 4 Gy (1h)
CE/A83B4C63 + 4 Gy (4h)
NP/A83B4C63 + 4 Gy (1h)
NP/A83B4C63 + 4 Gy (4h)
PARP
Cleaved PARP
β-actin
Caspase-7
Cleaved Caspase-7
β-actin
Caspase-3
Cleaved Caspase-3 (19 kDa)
Cleaved Caspase-3 (17 kDa)
β-actin

B

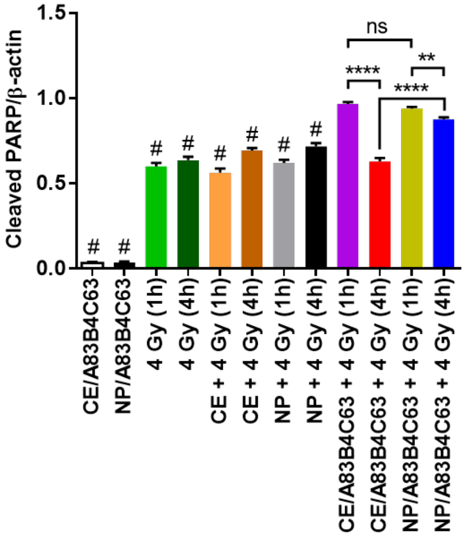

Cleaved PARP/β-actin
1.5
1.0
0.5
0.0
ns
****
**
****
#
CE/A83B4C63
NP/A83B4C63
4 Gy (1h)
4 Gy (4h)
CE + 4 Gy (1h)
CE + 4 Gy (4h)
NP + 4 Gy (1h)
NP + 4 Gy (4h)
CE/A83B4C63 + 4 Gy (1h)
CE/A83B4C63 + 4 Gy (4h)
NP/A83B4C63 + 4 Gy (1h)
NP/A83B4C63 + 4 Gy (4h)

C

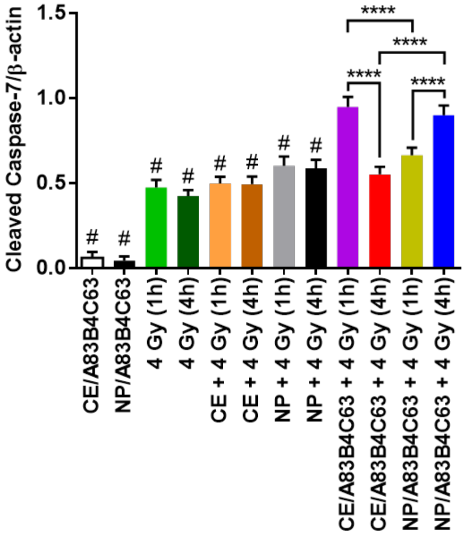

Cleaved Caspase-7/β-actin
1.5
1.0
0.5
0.0
****
****
****
****
#
CE/A83B4C63
NP/A83B4C63
4 Gy (1h)
4 Gy (4h)
CE + 4 Gy (1h)
CE + 4 Gy (4h)
NP + 4 Gy (1h)
NP + 4 Gy (4h)
CE/A83B4C63 + 4 Gy (1h)
CE/A83B4C63 + 4 Gy (4h)
NP/A83B4C63 + 4 Gy (1h)
NP/A83B4C63 + 4 Gy (4h)

D

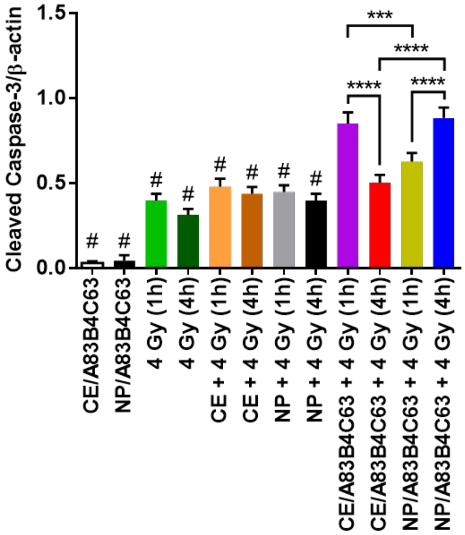

Cleaved Caspase-3/β-actin
1.5
1.0
0.5
0.0
***
****
****
****
#
CE/A83B4C63
NP/A83B4C63
4 Gy (1h)
4 Gy (4h)
CE + 4 Gy (1h)
CE + 4 Gy (4h)
NP + 4 Gy (1h)
NP + 4 Gy (4h)
CE/A83B4C63 + 4 Gy (1h)
CE/A83B4C63 + 4 Gy (4h)
NP/A83B4C63 + 4 Gy (1h)
NP/A83B4C63 + 4 Gy (4h)

Figure S2- (A) Representative western blot detection of cleaved PARP, cleaved caspase-7, and cleaved caspase-3 in HCT116 CRC cells pretreated with or without PNKP inhibitor (either CE/A83 or NP/A83 formulation). β-actin was used as a loading control. The conditions for all sample preparations and western blots were the same. The statistical analysis for the protein levels of (B) cleaved PARP, (C) cleaved caspase-7, and (D) cleaved caspase-3 was performed after normalization to β-actin. Differences were considered significant if $^{*}p \leq 0.05$, $^{**}p \leq 0.01$, $^{***}p \leq 0.001$, and $^{****}p \leq 0.0001$ following two-way ANOVA followed by Tukey's method. Data are expressed as mean ± SD (n = 3).
